# Assessment of post-abortion contraceptive counseling and practices among healthcare providers in Mfoundi Division, Centre Region, Cameroon

**DOI:** 10.11604/pamj.2025.52.69.47404

**Published:** 2025-10-14

**Authors:** Aïcha Celess Dongmo Megnidong, Olubukola Adeponle Adesina, Noel Vogue, Florent Ymele Fouelifack

**Affiliations:** 1 Reproductive Health Program, Pan African University Life and Earth Sciences Institute (including Health and Agriculture), Ibadan, Oyo State, Nigeria; 2 Department of Obstetrics and Gynecology, College of Medicine, University of Ibadan, University College Hospital, Ibadan, Oyo State, Nigeria; 3 Department of Public Health, Regional Delegation of Public Health, Center Region, Yaounde, Cameroon; 4 Department of Obstetrics and Gynecology, Central Hospital Yaounde, Yaounde, Cameroon

**Keywords:** Post-abortion care, family planning services, healthcare workers, contraception, Cameroon

## Abstract

**Introduction:**

post-abortion contraception, when administered before hospital discharge, effectively prevents unintended pregnancies and subsequent abortions. This study assessed the proportion of healthcare providers offering post-abortion contraceptive services in the Mfoundi Division of Cameroon and identified factors influencing these practices.

**Methods:**

we conducted a hospital-based cross-sectional study among 332 healthcare providers offering post-abortion care from 21 health facilities (10 private and 11 public) in the Mfoundi Division. Using a non-probabilistic sampling method, we selected two-thirds of participants from public facilities and one-third from private facilities. A pretested structured questionnaire was used for data collection, and SPSS 23.0. For data analysis. Binary logistic regression (p<0.05) determined associated factors.

**Results:**

among the participants were obstetricians/gynecologists (4.9%), midwives (44.6%), nurses (27.7%), general practitioners (9.6%), junior residents (6.9%), and senior residents (4.2%). We had 81.63% offering post-abortion contraceptive counseling, whereas 33.1% offered contraceptive methods prior to hospital discharge. Post abortion contraceptive counseling was associated with training on family planning (OR 3.8, p<0.001) and on post-abortion care (PAC) (OR 4.5, p=0.002). The non-availability of contraceptives (OR 0.18, p<0.001), knowing the importance of contraceptives during PAC (OR 2.6, p<=0.001), being an obstetrician/gynecologist (OR 1.3, p=0.012), family planning training (OR 3.8, p=0.003), and the availability of protocols (OR 2.8, p<0.001) were associated with post-abortion contraceptives provision.

**Conclusion:**

these results highlight the necessity of enhancing the post-abortion contraceptive practices in health facilities.

## Introduction

Abortion is the termination of a pregnancy before fetal viability, usually at 20 weeks of gestation or when the fetus weighs 500 grams or less [1]. Unsafe abortion is termination performed by unqualified individuals and/or in non-medical settings [2]. Africa has a high percentage of an active youthful population, characterized by high fertility rates, low contraceptive prevalence, and elevated maternal mortality rates [3]. Restricted access to effective pregnancy regulation and the criminalization of safe abortion contribute to unsafe abortion, raising maternal mortality in Africa [3]. Each year, over 3.4 million induced abortions occur in Africa; despite legal restrictions in many sub-Saharan nations, 35% of young adult pregnancies end in abortion, and up to 19% of adolescents report multiple abortions [4-6].

At the 1994 International Conference on Population and Development (ICPD), 179 nations, including those in sub-Saharan Africa, committed to prevent unsafe abortion and strengthen post-abortion care (PAC), emphasizing contraceptive counseling and provision regardless of abortion´s legal status [2,7].

Post-abortion care follows a five-component model: treatment of complications, counseling and family planning, contraceptive provision, referrals, and community interventions, and is delivered by a range of providers [8]. Since fertility returns quickly after abortion, contraceptive provision during PAC is vital to prevent repeat unintended pregnancies and unsafe abortion [9]. WHO recommends that all trained providers should offer counseling and contraception before discharge [10]. The prevalence of post-abortion contraceptive use varies significantly, ranging from 20.5% to 92%, with higher use when counseling is provided, methods are available on-site, and providers are trained [8,9,11-14].

Barriers to post-abortion contraceptive counseling include restrictive laws, poor service integration, institutional constraints, stigma, costs, and weak provider-patient relationships [8,15]. In Cameroon, 36% of unintended pregnancies end as unsafe abortion, and 40% of women aged 20 and above report at least two abortions in their lives [16]. Many PAC clients are motivated to use contraception but may not return for follow-up contraception services, making immediate counseling crucial during PAC [17].

World Health Organization, United Nations Population Fund, and International Federation of Gynecology and Obstetrics support widespread PAC contraceptive counseling to reduce maternal morbidity, mortality, and health system costs [18]. However, little is known about the providers´ role in Cameroon. This study assessed the proportion of healthcare providers offering counseling and methods in the Mfoundi Division, identified provider characteristics associated with these services, and reported barriers to their delivery.

## Methods

**Study design:** this study was a hospital-based cross-sectional study.

**Study area and setting:** this study was conducted in the obstetrics and gynecology services of private and public health facilities in the Mfoundi division, located in the Center region of Cameroon, from October to December 2024 (3 months). Cameroon is a lower-middle-income country in Central Africa, divided into 10 regions and 58 divisions. The Center Region, where the Mfoundi Division is located, has the highest number of healthcare facilities and personnel, making it a strategic location for studying PAC services. According to the National Institute of Statistics, the Center Region is the region in Cameroon with the highest number of health structures and healthcare personnel. The personnel-to-population ratio per 1000 across the regions in Cameroon varies significantly, ranging from a high of 2.0 to a low of 0.2. The Center Region stands out as the only region with a health personnel-to-population ratio of 2.0 per 1000 [19]. It is a cosmopolitan area and encompasses 10 divisions (out of 58 nationwide), 32 health districts, and one urban community (Yaoundé). There are notable differences in population densities between the 10 divisions in the central region. With a population density of 6,336.3 people per square kilometer, the Mfoundi Division is the most densely populated division in the central region and in the country due to the rapid population settlement, especially following the economic crisis of the 1980s.

The Cameroonian public health system is organized hierarchically. It follows a pyramidal structure with a centralized system of administration. Administration starts at the central level (strategic level), moves through intermediary regional delegations (technical level), and ends at the peripheral level (operational level), which encompasses the health districts. Healthcare facilities are of three levels (central, intermediate, and peripheral) and categorized as follows: from the ministerial classification, we have: 1) category 1: general hospitals and referral hospitals (tertiary level); 2) category 2: central hospitals (tertiary level); 3) category 3: regional hospitals and equivalents (secondary level); 4) category 4: district hospitals and equivalents (primary level); 5) category 5: CMA (medicalized health centers, primary level); 6) category 6: CSI (integrated health centers, primary level).

The center region comprises 32 health districts. Within these districts, we have several hospitals classified in categories. Mfoundi includes four hospitals that are categorized as category 1 (out of four in the central region), three that are categorized as category 2 (out of 3 in the central region), no hospital that is categorized as category 3 (out of 1 in the central region), eight that are categorized as category 4 (out of 31 in the central region), eleven that are categorized as category 5 (out of 51 in the central region), and twelve that are categorized as category 6 (out of 369 in the central region). The classification of health facilities into categories. Private health institutions, including non-profit religious associations as well as non-governmental organizations (NGOs) and for-profit institutions, operate alongside public facilities but are not formally categorized. However, some private facilities are well-equipped and offer services comparable to those of higher-category public hospitals; nonetheless, they are not formally classified. Among these facilities, many offer post-abortion care services in both the public and private sectors.

**Selection criteria:** we enrolled consenting healthcare providers working in the obstetrics and gynecology departments of selected health facilities, specifically those directly involved in post-abortion care, and who consented to participate in the study. Providers who were unavailable at the time of data collection were excluded.

**Sample size calculation:** we used the Cochran formula [20] for sample size calculation.


$$n_{0}=\frac{z^{2}pq}{e^{2}}$$

Where Z: the standard normal variant at a 95 % confidence level = 1.96; p: the expected proportion (0.3); q: (1-p); e: accuracy of the measurement = 0.05; n_0_: minimum sample size.

Since we had difficulty finding studies in central African countries directly assessing the proportion of healthcare providers offering contraceptive counseling during post-abortion care, we used a prevalence estimate of 30% from a study conducted in Tanzania [21]. While Tanzania and Cameroon differ in certain aspects of healthcare delivery, they are facing similar challenges related to post-abortion care integration, providers' training, and access to post-abortion contraception. In the absence of local data, this estimate was considered a reasonable approximation. After calculation, the minimum required sample size for this study was 323.

**Sampling technique:** we employed a convenient non-probabilistic sampling approach. Due to logistical constraints and the voluntary nature of the response, we adopted a non-probability convenience sampling approach. Despite this method may introduce selection bias, we minimized this by ensuring a diverse representation of providers across public and private health facilities in the Mfoundi.

In Cameroon, approximately 2/3 of deliveries take place in health facilities, with the remaining 1/3 of births occurring at home. Among the percentage, approximately 45% (2/3) of pregnant women deliver in public health facilities, while 23% (1/3) deliver in private facilities [22]. Given the fact that public hospitals handle a larger proportion of pregnant women, they are more likely to manage post-abortion care cases compared to private hospitals; therefore, we sampled two-thirds of our participants from public facilities and one-third from private facilities. The public health facilities in Cameroon, classified as categories 5 and 6, usually focus on basic health services. They have minimal patient inflow and a limited number of healthcare providers. In addition, cases requiring PAC are typically referred to higher-tier facilities (categories 1-4), where more specialized services and more personnel are available [19]. These lower-level facilities would not have significantly contributed to understanding PAC family planning services. We focused on public institutions (categories 1-4) offering post-abortion care in the Mfoundi Division, and to ensure representativeness of the private sector, we consecutively included private health facilities providing post-abortion care until we reached our target sample size. Within each selected healthcare facility, we identified key informants who are directly involved in providing post-abortion services. These informants included gynecologists/obstetricians, nurses, midwives, internists/residents, and general practitioners as applicable.

**Study variables:** the primary outcome measures were the provision of post-abortion contraceptive counseling and method provision, which were assessed via questionnaire items with yes/no responses. Counseling was defined as a discussion of contraceptive methods, effectiveness, side effects, and patient preference by a provider. Provision was defined as the provision or prescription of a contraceptive method. Independent variables included demographics, professional knowledge (PAC training, experience), and facility attributes. This includes discussion of the effectiveness, benefits, risks, and side effects of different contraceptives, as well as helping them choose the most suitable method based on their needs and preferences. Contraception provision was referred to as any deliberate provision of methods, techniques, or devices to prevent pregnancy. Independent variables included socio-demographic variables, health provider qualifications, professional knowledge, professional practice, factors acting as barriers, and facilitator variables.

**Data collection tools:** we used a structured questionnaire as our data collection tool, which was adapted from validated instruments that have been successfully employed in similar studies. To ensure everything was on point, we had experts review for content validity. The questionnaire consisted of 4 domains in addition to identification: (1) socio-demographic data, (2) professional qualification and knowledge on post abortion care of PAC, (3) contraceptive practices during PAC, and (4) perceived barriers to the delivery of PAC services. Before data collection, the questionnaire was content-validated by experts and piloted among 10 providers to check for clarity and reliability, and make it simple and understandable. Questionnaires were prepared in English and translated into French. The questionnaire was content-validated by experts and piloted among 10 providers to make it simple and reliable.

**Data analysis:** to ensure confidentiality, the data retrieved were anonymized. The questionnaires were cross-checked daily to ensure completeness of information recorded, and when incomplete, the questionnaire was removed and another one was filled out; we reached our minimum sample size. Data were entered into CS Pro version 7.0 and exported to the Software Statistical Package for Social Sciences (SPSS) version 23.0 for analysis. We summarized categorical variables like provider qualifications and facility types as frequencies and percentages. For continuous variables, such as the years of experience, we checked for normality and reported the data as means with standard deviations for those that were normally distributed, or as medians with interquartile ranges for those that were skewed. To explore associations, we used chi-square tests for categorical variables and either t-tests or Mann-Whitney U tests for continuous variables, depending on what was appropriate. We then used binary logistic regression, making sure to adjust for any potential confounding factors, with a p-value <0.05 considered statistically significant. Results were represented in tables and figures (pie charts, bar charts, and tables) to ease organization and comprehension.

**Data availability statement:** the data that support the findings of this study are available from the corresponding authors upon reasonable request.

**Ethical consideration:** after obtaining ethical clearance from the UI/UCH Ethics Review Committee, Nigeria (Ref. No UI/EC/24/0488), and from the Ethics Committee for Research in Human Health of the Central Region, Cameroon (Ref. No 01053/CRERSHC/2024), we obtained administrative authorizations from the directors of the hospitals involved and introduced ourselves to the heads of departments of health facilities. Following ethical clearances and administrative authorizations, we started data collection from eligible, consenting healthcare providers. In order to ensure confidentiality of information, the questionnaires were anonymous and coded; no names were taken. To ensure the privacy of participants, data was collected individually rather than collectively. To ensure participants fully understand the study, the participant information sheet, consent forms, and questionnaire were made in both English and French. There was no risk to the participants of the study, as well as no repercussions for those whose records were used for the study. No harmful practice was done, nor was an invasive procedure performed, as the study only requires the completion of questionnaires. Informed consent was obtained from all participants, ensuring they were fully aware of the study´s purpose, procedures, potential risks, and benefits. The involvement of participants was entirely voluntary, and they were able to withdraw at any time without any repercussions.

## Results

We recruited a total of 221 (66.6%) eligible participants from 11 public facilities and 111 (33.4%) participants from 10 private health facilities.

**Socio-demographic characteristics and professional qualifications of the study population:** as shown in Table 1, the mean age (SD) of participants was 32.9 (7.9) years, with 49.4% (164) of the participants aged between 30-39 years. Out of the 332 participants included, 79.5% (264) were female, and 59.3% (197) identified as belonging to the Catholic religion. One (1) of the participants was from Nigeria, two (2) from the Central African Republic, and one (1) from the DRC. The majority of participants (44.6% (148)) were midwives. The mean (SD) years of experience were 6 (6.5) years.

**Table 1:** demographic and professional characteristics of healthcare providers involved in post-abortion contraceptive counseling and practices in Mfoundi, Centre Region, Cameroon (N=332)

Variables		
Age range (years)	Proportion(n)	Frequency (%)
20-29	122	36.7
30-39	164	49.4
40-49	22	6.6
50-59	24	7.2
60 years and above	1	0.3
**Religion**		
Protestant	83	25,0
Catholic	197	59.3
Muslim	20	6.0
Atheist	5	1.5
Pentecostal	24	7.2
**A cadre of health professionals**		
Obstetrician/Gynecologist	23	6.9
Senior resident (3^rd^ and 4^th^ year)	14	4.2
Junior resident (1^st^ and 2^nd^ year)	23	6.9
General practitioners	32	9.6
Midwife	148	44.6
Nurse	92	27.7
**Years of experience as a healthcare provider**		
≤ 2	114	34.3
3 - 5	107	32.2
6 - 10	67	20.2
>10	44	13.3
**Received training on post abortion care**		
Yes	218	65.7
No	114	34.3
**Availability of protocol or guideline in the health center**		
Availability of a formal protocol that is actively used	38	14.4
Availability of a formal but it is not regularly followed or referenced	114	34.3
Non-availability of a protocol, but in the process of developing one	30	9.0
Non-Availability of a formal plan to develop one	150	45.2
**Ever received training on family planning**		
Yes	316	95.2
No	16	4.8
**Practitioners think it is important to provide modern contraceptives during post abortion care**		
Yes	300	90.4
No	32	9.6

**Professional knowledge and professional practice of the study population:** as it is still shown in Table 1, out of 332 participants, 218 (65.7%) received training on post-abortion care, with 95.5% (317) and 78.9% (262) of health personnel recognizing family planning counseling and contraceptive provision, respectively as part of post-abortion care. Among the 332 participants, 313 (95.2%) reported having received training on family planning, mostly on injectable contraceptives (87.9%; 292) and implants (87.9%; 292). Out of the 332 participants, 99.4% (330) think it is important to provide family planning counseling during post-abortion care, and 90.4% (300) think it is also important to provide contraceptive methods before hospital discharge. The median (25^th^-75^th^) of patients received for post abortion care on average in a month was 7 [5-10], with a range between 2 to 35 patients per month. The most recommended contraceptive methods were implants 59.6% (198), followed by condoms 54.5% (181), and injectable contraceptives 41.9% (138).

**Figure 1:**
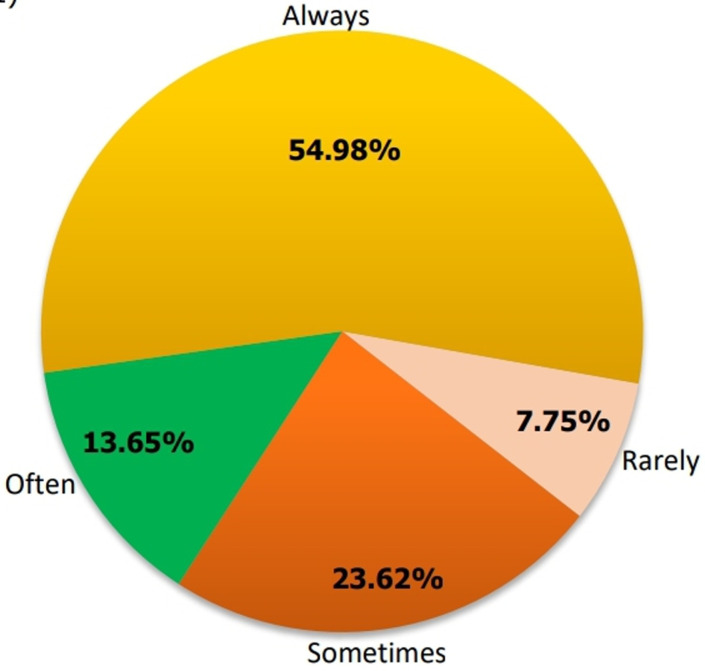
frequency of contraceptive counseling delivered by healthcare providers during post-abortion care in Mfoundi, Centre Region, Cameroon (N=271)

**Proportion of health care providers who offer comprehensive post-abortion contraceptive counseling in the Mfoundi Division:** out of the 332 participants, 271 (81.6%) reported offering contraceptive counseling as part of post-abortion care. Of the 271 participants who offered counseling, 54.9% (142) always included family planning counseling, as represented in Figure 1.

**Characteristics of the study population associated with post-abortion contraceptive counseling (binary logistic regression):** as shown in Table 2, after adjustment for variables with significant p-value on bivariate analysis, Participants who received training on post-abortion care were 4.5 times more likely to provide contraceptive counseling compared to those without training (p=0.002, OR 4.5), and participants receiving training on family planning were 3.8 times more likely to offered counseling during post abortion care (p<0.001, OR 3.8).

**Table 2:** factors associated with post-abortion contraceptive counseling among healthcare providers in Mfoundi, Centre Region, Cameroon: results from binary logistic regression analysis

Variables	OR	95% CI	p-value
**Training on post-abortion care**			
No	-	-	-
Yes	4.5	1.84 - 11.15	0.002*
**Training on family planning**			
No	-	-	-
Yes	3.8	1.56 - 9.39	<0.001*
**Hospital policy on the provision of contraceptive during PAC**			
Contraceptives are automatically provided to all patients as part of post-abortion care	-	-	-
Contraceptives are provided only upon patient request	0.8	0.4 - 1.72	0.7
Provision of contraceptives is at the discretion of the attending healthcare provider	0.76	0.6 - 1.92	0.6
There is no specific policy; practices vary by department	0.69	0.5 - 2.62	0.9
OR: odds ratio; CI: confidence interval; PAC: post-abortion care			

**Figure 2:**
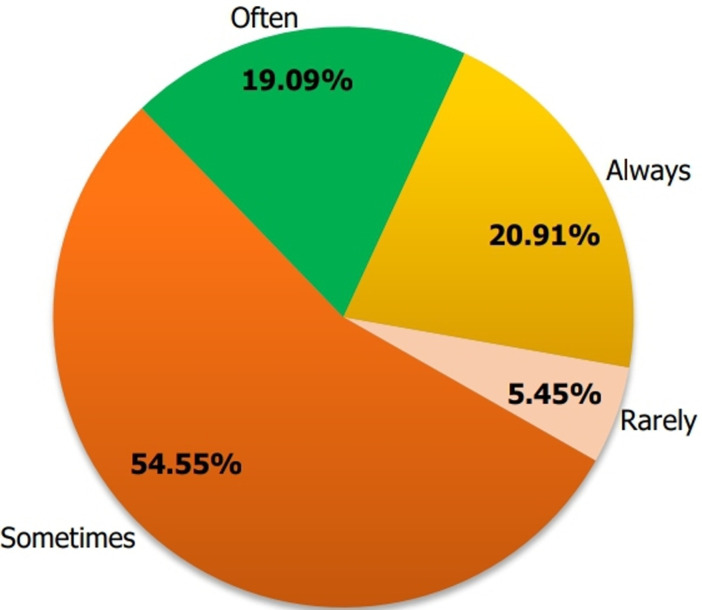
frequency of contraceptive method provision by healthcare providers during post-abortion care in Mfoundi, Centre Region, Cameroon (N=110)

**Proportion of participants providing contraceptive methods before discharge from the hospital during post-abortion care:** a total of 110 (33.1%) healthcare providers out of the 332 participants declared providing contraceptive methods before hospital discharge as part of post-abortion care. Of the 110 participants providing contraceptive methods, 20.9 % (n=23) reported always providing contraception before hospital discharge, as represented in Figure 2.

**Participants' characteristics associated with post-abortion contraceptive methods provision (binary logistic regression):** as shown in Table 3, after adjustment for variables with significant p-values on bivariate analysis, obstetricians and gynecologists were 1.3 times more likely to provide contraceptives compared to general practitioners (p=0.012, OR 1.32). Healthcare providers with a clear, available protocol in the services were 2.8 times more likely to provide contraception compared to those without a formal protocol actively used (p<0.001, OR 2.8). Training on family planning (p=0.003, OR 3.8), knowing that it is important to provide contraceptives during post-abortion care (p=0.001, OR 2.6), and the non-availability of contraceptives in the obstetrics and gynecology service (p<0.001, OR 0.18) were also significantly associated with post-abortion contraceptive provision before hospital discharge.

**Table 3:** factors associated with post-abortion contraceptive provision among healthcare providers in Mfoundi, Centre Region, and Cameroon: results from binary logistic regression analysis

Variables	OR^1^	95% CI^1^	p-value
**Professional qualification**			
General practitioner	-	-	-
Nurse	0.8	0.36 - 4.88	0.7
Midwife	0.91	0.26 - 3.22	0.9
Junior resident (first and second year)	1.01	0.19 - 4.93	0.2
Senior resident (third and fourth year)	1.09	0.05 - 3.29	0.4
Obstetrician/Gynecologist	1.32	0.61 - 2.8	0.012*
**Availability of protocol**			
No, we do not have a protocol, and there are no current plans to develop one	-	-	-
No, we do not have a protocol, but we are in the process of developing one	1.05	0.65 - 5.05	0.2
Yes, we have a formal protocol that is actively used	2.88	1.67 - 4.96	<0.001*
Yes, we have a protocol, but it is not regularly followed or referenced	1.58	0.55 - 6.17	0.1
**Training on family planning**			
No	-	-	-
Yes	**3.83**	1.56 - 9.39	0.003*
**Importance of providing contraceptives before hospital discharge during PAC**			
No	-	-	-
Yes	2.6	1.51 - 6.72	0.001*
**Availability of contraceptives in the services**			
All methods available	-	-	-
No methods available	0.18	0.07 - 0.49	<0.001*
^1^OR: odds ratio; CI: confidence interval; PAC: post abortion care			

**Factors acting as barriers to post-abortion contraceptive provision:** as shown in Table 4, out of 332 participants, 51.5% (171) reported not having contraceptives available in their services, leading to the referral of patients to family planning services. However, there was no reported follow-up mechanism to verify whether patients eventually received contraception. Six percent of participants (6%, n=20) reported having a strict hospital policy concerning contraceptive practices during post-abortion care. While 91.9% of participants reported appropriate skills for the administration of injectable contraceptives, 37.7% were trained in IUD insertion. Despite the availability of time (85.2%) and place (97.9%) for post-abortion contraceptive practices, lack of contraceptive availability remains a key barrier.

**Table 4:** factors reported as barriers to post-abortion contraceptive practice among healthcare providers in Mfoundi, Centre Region, Cameroon (N=332)

Variables	Proportion(n)	Frequency (%)
**Availability of contraceptives in the services**		
At least one method was available	148	44.6
All methods available	13	3.9
No method available	171	51.5
**Hospital policy regarding the provision of contraceptives during post-abortion care**		
Automatic provision	20	6.0
Contraceptive provision based on provider	219	66.0
Provision upon patient request.	2	0.6
No policy	91	27.4
**Availability of a private place for counseling**		
Yes	322	97.0
No	10	3
**Availability of a private place for contraceptive provision**		
Yes	325	97.9
No	7	2.1
**Having time for contraceptive counseling**		
Yes	292	88.0
No	40	12
**Having time for contraceptive provision**		
Yes	283	85.2
No	49	14.8
**Skills to provide contraceptive methods**		
IUD	125	37.7
Injectable contraceptives	305	91.9
Implants	241	72.6
Pills	236	71.1
Condom	323	97.3
Sterilization	34	10.2
IUD: intrauterine device		

## Discussion

This study looked into how healthcare providers in Mfoundi, Cameroon, practice post-abortion contraceptive counseling and method provision. Our results show that while 81.6% of providers offered counseling on family planning after an abortion, only 33.1% actually provided contraceptive methods before hospital discharge. We found that key factors affecting whether counseling was given included whether the providers had received prior training in post-abortion care (those who had were 4.5 times more likely to offer counseling, (p=0.002)), and training on family planning (OR 3.8, p<0.001). The main barrier to the provision of contraceptives was the non-availability of contraceptives (OR 0.18, p<0.001) and the absence of standardized protocols in healthcare facilities (OR 2.8, p<0.001). The inclusion of healthcare professionals from a range of public and private facilities is a significant strength in this study, as it helps to clarify the situation in various healthcare settings in the Mfoundi Division of Cameroon. However, we were limited by the cross-sectional design of the study, making definitive conclusions regarding cause and effect difficult to visualize. Furthermore, because the study relied on self-reported data, participant responses may have been biased.

When compared with similar studies in other countries, the results from this study show both similarities and differences. For example, 81.6% of providers in this study offering post-abortion counseling is higher than the rates found in Egypt [14], Ethiopia [23], and Kenya [24]. This may be attributed to the differences in provider training. For example, in Ethiopia, only 23% of providers had post-abortion care training, compared to 65.5% in our study. However, our findings are closer to the 88.8% reported in Nigeria [13], though they are lower than the 92% reported in Kenya in the study by Mbehero *et al*. [25]. These differences might be due to variations in healthcare provider training and differences in the study designs. For example, 65.5% of the healthcare providers in our study received training on post-abortion care, while only 23% were trained in Ethiopia [23]. This could explain why the counseling and contraceptive provision were different. Moreover, studies like Mbehero *et al*. in Kenya, which involved more extensive provider training, reported higher provision rates [25].

This study supports previous findings that improved post-abortion contraceptive counseling and provision require well-elaborated and clear protocols, access to contraceptive methods at all times, and continuous training for healthcare providers [8,9,11-14,17]. Training increases the confidence level of healthcare professionals, enabling them to offer more accurate counseling during post-abortion care, when patients may be more open and receptive to contraceptive options.

Our study found that only 33.1% of healthcare providers offer contraceptives before discharge. This is lower than rates reported in structured programs, such as those in Janie *et al*. [9]. The relatively low rate of contraceptive provision before hospital discharge in our study, which stands at just 33.1%, could be attributed to a few significant factors. There may be a lack of clear institutional guidelines for post-abortion contraception, inadequate training for healthcare providers, and issues within the supply chain. Without clear protocols, healthcare providers might hesitate to offer contraceptives immediately after an abortion, leading to missed opportunities for preventing future pregnancies. Research from Kenya and Ethiopia shows that when structured protocols for post-abortion contraception are implemented, the uptake can exceed 50% [15,16]. Post-abortion contraceptive provision can be increased by enhancing provider education and guaranteeing contraceptive availability in the services, as well as the availability of a clear protocol.

Our research found that being an OB/GYN, following a formal protocol, receiving family planning training, understanding the significance of contraception in PAC, and having access to contraceptives are all important factors linked to the provision of post-abortion contraception. These findings are corroborated by Benson *et al*. as well as Owolabi *et al*. who highlight the importance of training and contraceptive availability as key elements to the provision of contraception prior to hospital discharge [17,26]. However, some studies did not find an association with provider qualification [27]. This difference may be due to variations in study settings; in our context, OB/GYNs have more decision-making power, and strengthening training and protocol adherence remains crucial for improving services.

Several barriers have been found to limit the effective provision of post-abortion contraceptives. One of the major challenges is the unavailability of contraceptives directly in the services, with over half of providers reporting stockouts. Although most providers could administer injectable contraceptives, only 37.7% reported having the skill to place IUDs, reflecting a gap in Long-Acting Reversible Contraceptives (LARCs) skills. Previous studies confirm that limited provider skills hinder LARC provision [14,26]. While time and space were reported by participants to be sufficient, the lack of contraceptive methods remains a major obstacle. Addressing supply chain issues and enhancing provider training are essential for improving post-abortion contraceptive access in our context.

**Limitations:** this study is limited by its quantitative design, which may not have captured the full complexity of provider attitudes and behaviors towards post-abortion contraception and perceived barriers. Additionally, the sample size calculation, based on the reported prevalence derived from a study done in Tanzania due to limited local data, may not have been the most precise for the local context.

## Conclusion

The findings of the study identified enablers and barriers to improve the quality of post-abortion family planning counseling and contraceptive provision in the Mfoundi division of the Center Region of Cameroon. We found that only 54.9% of healthcare providers always offer post-abortion family planning counseling out of the 81.6% who reported provision of contraceptive counseling during post-abortion care, and 33.1% offer contraceptive methods prior to hospital discharge. The provision of post-abortion contraceptive methods was substantially associated with factors such as family planning training, the availability of contraceptive methods in the services, and understanding the significance of post-abortion contraceptive provision during post-abortion care. To enhance post-abortion contraceptive practices, we need solid policy backing, proper resource distribution, and ongoing training for healthcare providers. By making sure contraceptives are readily available and putting standardized protocols in place, we can significantly improve family planning services after an abortion and help lower the rates of unintended pregnancies and unsafe abortion.

### What is known about this topic

- Post-abortion family planning and contraceptive utilization can educate women and their partners on the fact that family planning measures can save their time, morbidity, and resources;
- Post-abortion contraceptive uptake is high when contraceptive counselling is done and delivered at the time of post-abortion care, when contraceptive methods are readily available in the post-abortion care room, when healthcare providers are trained and offered comprehensive information, and when there is place and time available for contraceptive practice during post-abortion care;
- Factors acting as a barrier for post- abortion contraceptive counseling among health care providers include policy and legal restrictions on both abortion and contraception; the lack of administrative coordination between abortion care and contraceptive services further hinders effective post-abortion contraceptive provision.

### What this study adds

- In Mfoundi, only 54.9% of healthcare providers always offered post-abortion family planning counseling, while 33.1% provided contraceptive methods before discharge;
- Contraceptive provision during PAC was significantly more likely when providers had received family planning training, when contraceptives were available in the service, and when providers recognized the importance of contraceptive provision;
- Few healthcare providers had contraceptives or standardized protocols directly available at the point of PAC, which limited their ability to provide these services effectively.
